# Leveraging oncovirus-derived antigen against the viral malignancies in adoptive cell therapies

**DOI:** 10.1186/s40364-024-00617-6

**Published:** 2024-07-29

**Authors:** Wei Zhang, Miao Zeng, Yisheng Li, Li Yu

**Affiliations:** 1 Department of Hematology and Oncology, Shenzhen University General Hospital, International Cancer Center, Hematology Institution of Shenzhen University, Shenzhen University Medical School, Shenzhen University, Shenzhen, Guangdong 518000 China; 2 Guangdong Key Laboratory for Biomedical Measurements and Ultrasound Imaging, National-Regional Key Technology Engineering Laboratory for Medical Ultrasound, School of Biomedical Engineering, Shenzhen University Medical school, Shenzhen, 518060 China; 3 Shenzhen Haoshi Biotechnology Co., Ltd, No. 155 Hongtian Road, Xinqiao Street, Bao’an District, Shenzhen, Guangdong 518125 China; 4 Haoshi Cell Therapy Institute, Shenzhen University, Shenzhen, China

**Keywords:** Viral malignancy, Oncovirus, Oncoprotein, Adoptive cell therapies (ACTs), Viral antigen

## Abstract

Adoptive cell therapies (ACTs) have revolutionized cancer immunotherapy, prompting exploration into their application against oncoviruses. Oncoviruses such as human papillomavirus (HPV), hepatitis B virus (HBV), hepatitis C virus (HCV), and Epstein-Barr virus (EBV) contribute significantly (12-25%) to human malignancies through direct or indirect oncogenic mechanisms. These viruses persistently or latently infect cells, disrupt cellular homeostasis and pathways, challenging current antiviral treatment paradigms. Moreover, viral infections pose additional risks in the setting of long-term cancer therapy and lead to morbidity and mortality. Virally encoded oncoproteins, which are tumor-restricted, immunologically foreign, and even uniformly expressed, represent promising targets for patient-tailored ACTs. This review elucidates the rationale for leveraging viral antigen-specific ACTs in combating viral-associated malignancies. On this basis, ongoing preclinical studies consolidate our understanding of harnessing ACTs against viral malignancies, underscoring their potential to eradicate viruses implicated in cancer progression. Furthermore, we scrutinize the current landscape of clinical trials focusing on virus-specific ACTs and discuss their implications for therapeutic advancement.

## Introduction

The International Agency for Research on Cancer (IARC) recognizes 7 major human viruses as direct oncogenic agents, including human papilloma virus (HPV), hepatitis B virus (HBV), hepatitis C virus (HCV), Epstein-Barr virus (EBV), Kaposi’s sarcoma-associated herpesvirus (KSHV), Merkel cell polyomavirus (MCPV), and human immunodeficiency virus type 1 (HIV-1) [1]. Additionally, cytomegalovirus (CMV) reactivation and infection are frequently observed in immunocompromised individuals such as transplant recipients or HIV-1 carriers [2], with mounting evidence suggesting CMV’s potential as an oncogenic virus [3–6].

**Fig. 1:**
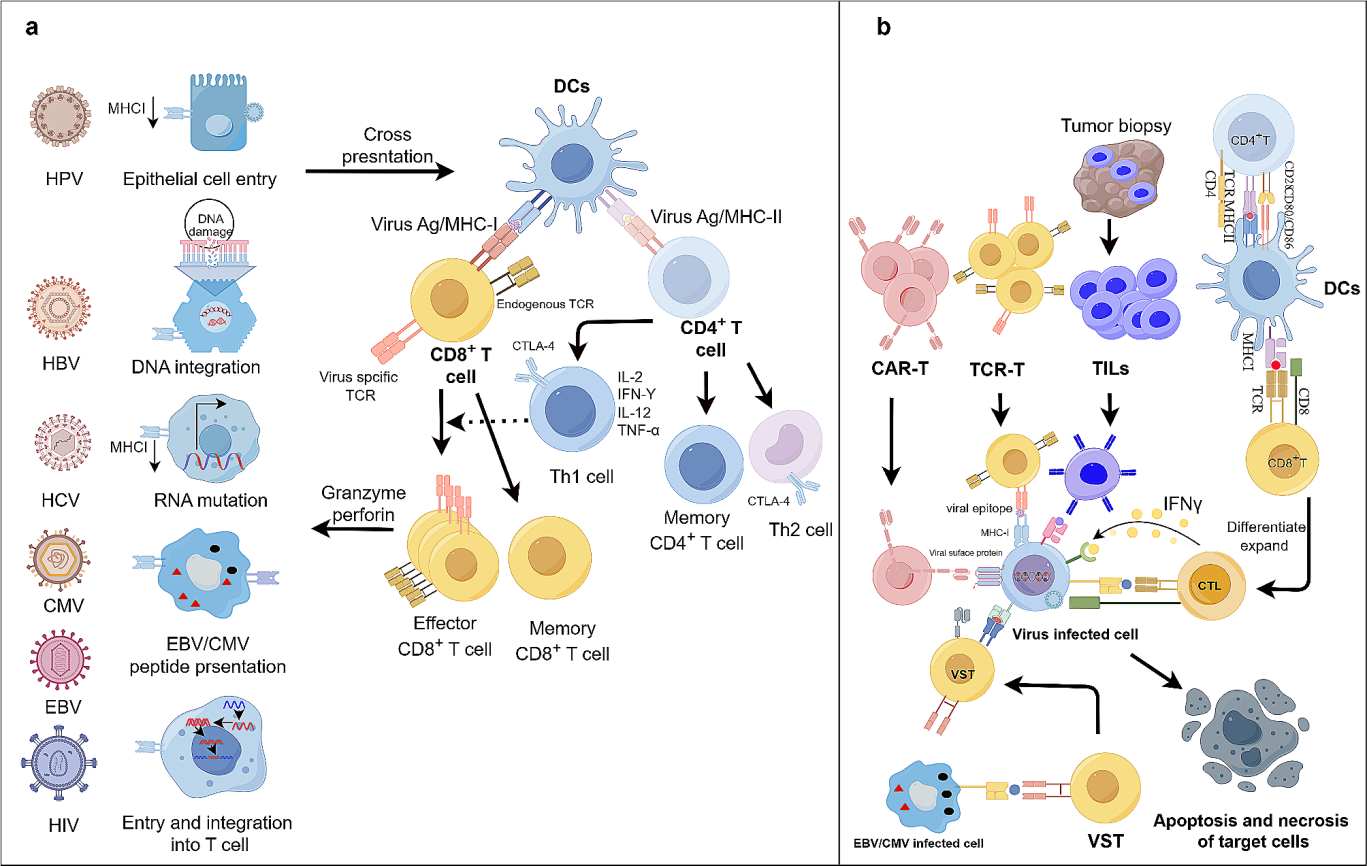
**a** Viral persistent or latent infection and host cellular immunity against oncogenic infection (By Figdraw). A variety of virally oncogenic mechanisms determine the transformation and maintenance of the malignancy. Virus-infected cells can be recognized and eliminated by host cellular immunity. **b** Schematic diagram of ACTs on viral malignancies. Engineered TILs, DCs, CAR-T cells, TCR-T cells, and VST therapies have the potential for application to viral malignancies. CAR-T cells target the virally-encoded cell surface antigen through an antibody-based scFv. In contrast, TCR-T cells target a virus-derived peptide on MHC complex. In TIL therapy, tumor is surgically resected and T-cells are expanded from the tumor ex vivo. TILs target viral antigens as well as non-viral proteins. The VST therapy aims to enhance the host immune system’s ability to clear infected cells by using activated virus specific T-cells. While the DCs enable to induce and amplify virus-specific CTLs.

Despite the typically robust immune response to viral antigens in most infected individuals, persistent or latent infection of oncoviruses enables to evade the immune system and induce immune tolerance through mechanisms such as downregulating major histocompatibility complex (MHC) molecules, producing immunosuppressive proteins, and directly infecting immune cells [7–9], which increases the risk of virus-driven or associated cancers (Fig. 1a). For instance, HPV, EBV and CMV encode viral oncoproteins that mimic or interfere with host regulatory mechanisms, disrupt cellular homeostasis, and impact cellular proteins, such as the tumor suppressor proteins p53 and pRb (Table 1) [2, 10]. In some other cases involving either DNA or RNA viruses (HPV, HBV, EBV, CMV and HIV-1), the viral genome can integrate into the host genome, remaining dormant until conditions favor reactivation, thereby contributing to viral persistence and disease manifestation [11]. Importantly, the “hit-and-run’’ theory also posits that viruses induce a series of cellular changes, promoting normal cells to become cancer cells, after which the virus leaves while the cancer cells develop [12]. However, HBV and HCV can create a microenvironment conducive to tumorigenesis through chronic inflammation. Persistent inflammation leads to the production of cytokines and growth factors, that promote cell proliferation, angiogenesis, and genomic instability [13].

**Table 1:** Carcinogenic traits and virus-specific adoptive cell therapies (ACTs) of various viral malignancies

Family/species	Virus feature	Related cancer	Tropism	Oncoproteins	Targeted antigen	ACTs
Papillomaviridae/HPV	Circular dsDNA 8 kb	CC, HNC, OPC, anal cancer, etc.	Epithelial cells	E2/5/6/7	E6, E7, L1	E7_11 − 19_:HLA CAR-T E7_11 − 19_ TCR-T, E6_92 − 101_TCR-T, E6_29 − 38_TCR-T HPV-TILs E7-DCs, L1-DCs
Hepadnaviridae/HBV	rcDNA 3.2 kb	HCC, intrahepatic cholangiocarcinoma, pancreatic cancer, colorectal cancer lymphoma, etc.	Liver cells, myocardial cells, skeletal muscle cells, lymphocytes, etc.,	HBx、HBsAg	HBsAg, HBcAg	SHB/LHB-CAR-T, HBsAg-CAR-T, preS1-CAR-T HBsAg_183 − 191_-TCR-T, HBsAg_370 − 379_-TCR-T HBsAg/HBcAg-TCR-T, HBsAg_171_-TCR-T, HBsAg_344 − 377_-TCR-T HBcAg_18 − 27_ and HBsAg_335 − 343−_loaded DCs HBsAg/HBcAg-pulsed DCs, HBVsvp-pulsed DCs TC-DCs
Flaviviridae/HCV	Linear (+) ssRNA 9.6 kb	HCC, B-NHL	Liver cells	NS3/4、NS5	E2, NS5, E2, NS3	HCV-E2-CAR-T, HCV-NS3-CAR-T NS3_1073 − 1081_-TCR-T, NS5_1992 − 2000_-TCR-T HCV-E2-DCs, HCV-NS3-DCs
Herpesviridae/CMV	Linear dsDNA 235-240 kb	Glioma, colorectal cancer, etc.	Macrophages, epithelial cells, endothelial cells, nerves, fibroblasts, immune cells	UL97、UL123	gB, pp65	CD19-CMV-CAR-T, Her2-CMV-CAR-T CMV-TCR-T, HA-CMV-TCR-T gB-CAR-T CMV-IE-DCs, CMV-pp65-DCs
Herpesviridae/EBV	DNA175kb	NPC, Hodgkin lymphoma, Burkitt’s lymphoma, T-cell lymphoma, gastric cancer, etc.	Epithelial cells, B/T cells	EBNA1、EBNA3、LMP1、LMP2、ncRNA、miRNA	LMP1, LMP2, gp350	LMP/EBNA1-CTL, LMP-CTL, BARF1-CTL, EBV-CTL LMP1-CD4^+^T Ad-ΔLMP1-LMP2-DCs, LMP2-DCs LMP1-CAR-T, gp350-CAR-T LMP1-TCR
Herpesviridae/KSHV (HHV8)	DNA 140 ~ 160 kb	Kaposi’s sarcoma	Epithelial cells B cells	LANA, vGPCR, vCYC, miRNAs and etc.		
Retroviridae/HIV-1	Linear (+) ssRNA 8-10 kb	B-NHL (DLBCL and Burkitt’s lymphoma), T-cell lymphoma	Monocytes/macrophages, T/B cells, CD4^+^helper T cells	Tax, HBZ, Tat, Rev, Nef, Vpr	gp41, gp120, Tat, Rev, Nef	CD4-CAR-T, bNAb-CAR-T, anti-HIV-1 duoCAR-T, anti-HIV-1 TCR-T, HIV-1-DC/DC-TRN
Polyomaviridae/MCPV	Circular dsDNA 5.4 kb	Merkel cell carcinoma	Merkel cells	T antigen		
Polyomaviridae/JCPV	Circular dsDNA 5.1 kb	Colon cancer	Lymphocytes, monocytes	T antigen		

Current antiviral treatments effectively suppress viral replication but fail to eliminate chronic or latent infections. Eradicating viral reservoirs remains a critical therapeutic challenge. Beyond tumors, virus-specific adoptive cell therapies (ACTs) have shown promise in purging viral infections, suggesting a potential role in treating viral malignancies [14]. ACTs are particularly suited to viral malignancies due to the expression of targetable tumor-associated viral antigens exclusively in cancerous cells, providing an unparalleled opportunity to subvert such oncoproteins as tumor-specific targets. Furthermore, the current tantalizing goal is to activate immune cells by targeting viral antigens, rejuvenate antiviral effects and achieve the goal of recognizing and killing virus-related tumor cells.

Tremendous progress has been made in the development of ACTs for viral malignancies, including tumor-infiltrating lymphocyte (TIL), dendritic cell (DC), chimeric antigen receptor (CAR)-T cell, T-cell receptor (TCR)-T cell, and virus-specific T-cell (VST) therapies (Fig. 1b), and these findings require reanalysis and reflection. Our review covers preclinical and clinical ACTs for the ablation of oncovirus infections and associated viral malignancies, highlighting the therapeutic potential of targeting virally encoded antigens.

## HPV

High-risk HPV types (HPV16/18) are well-established drivers of various cancer, including cervical carcinoma (CC), head and neck cancer (HNC) and oropharyngeal cancer (OPC) [15]. This oncogenic potential is primarily attributed to viral integration and oncoproteins [16–19]. The HPV genome encodes early (E1-E7) and late (L1 and L2) proteins during the viral life cycle. When the HPV genome gets integrated, constitutive E6 and E7 expression is observed, which is critical for the transformation and maintenance of malignancy by interfering with cellular homeostasis, inhibiting the immune response and inducing immune escape [20]. The pRb pathway is disrupted by the E7 protein, releasing the E2F transcription factor and leading to cell cycle dysregulation and unrestricted proliferation [21, 22]. E6 promotes p53 degradation, thus inhibiting p53-mediated apoptosis and facilitating an ongoing cell cycle for viral replication [23]. Multiple pathways, including the Wnt/β-catenin, Bak and PI3K/Akt pathways promote cancer progression by interfering with cell proliferation, differentiation, and apoptosis and inducing abnormal gene expression [24].

**Fig. 2:**
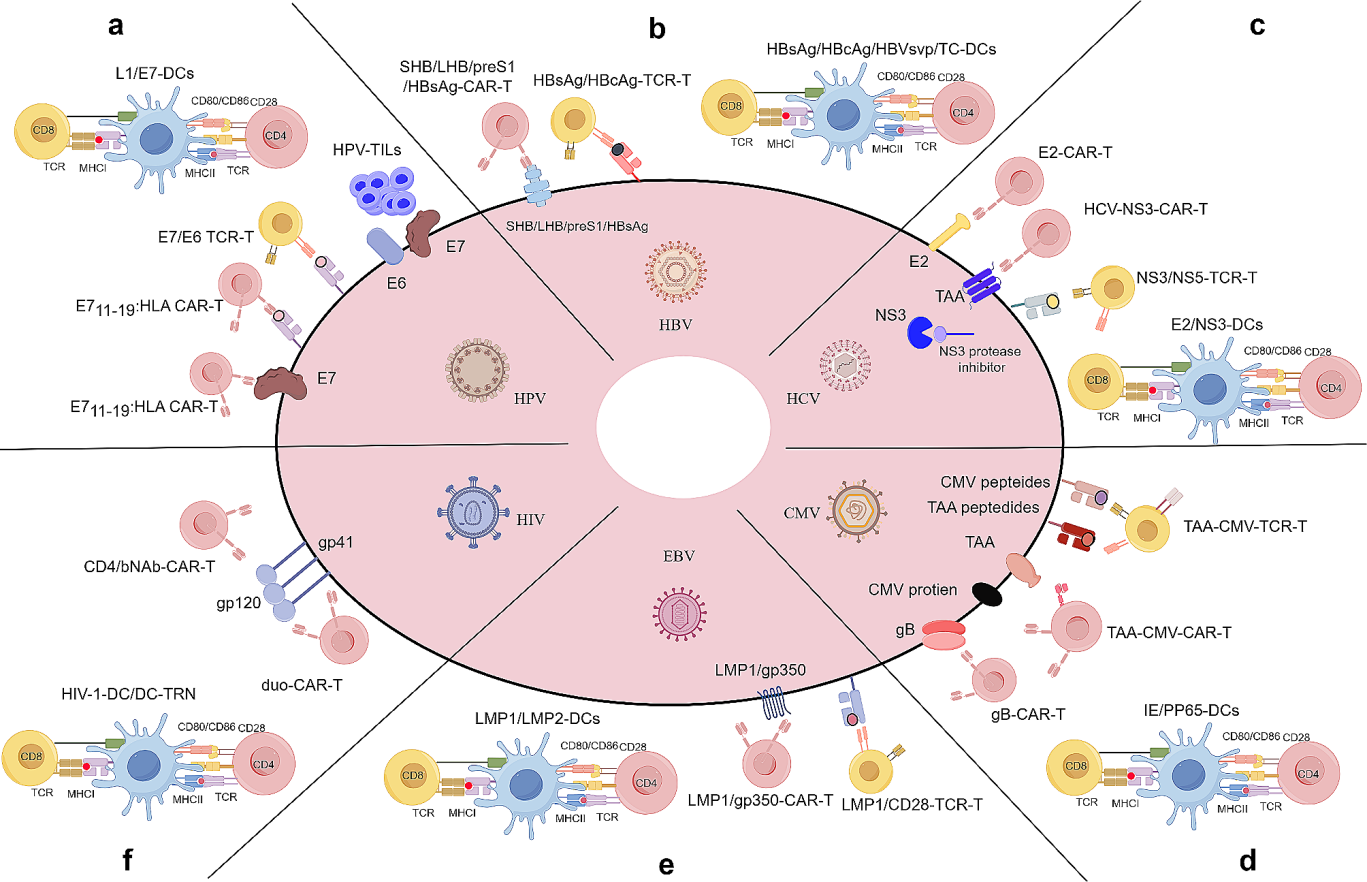
Virally encoded antigens and relevant utilization of oncovirus-specific ACTs in viral malignancies. **a** HPV-specific ACTs. **b** HBV-specific ACTs. **c** HCV-specific ACTs. **d** CMV-specific ACTs. **e** EBV-specific ACTs. **f** HIV-1-specific ACTs.

Even prophylactic vaccines are envisaged to protect immunized individuals against cancer-associated HPV genotypes. For established HPV infection or maintenance in a latent or asymptomatic state in basal cells, where the HPV integrates with the host cell genome and no longer expresses viral L1/2 antigens, the conventional preventive HPV vaccines have been demonstrated to be ineffective [25]. In contrast, therapeutic HPV vaccines focusing on HPV primary oncoproteins, specifically E6 and E7, represent a promising avenue for enhancing clinical outcomes among advanced-stage and recurrent patients without eliciting autoimmune or severe adverse events (Fig. 2a; Table 2). Notwithstanding, it is crucial to acknowledge that the most frequently encountered severe toxicities primarily manifest as hematologic complications, which are anticipated sequelae of lymphocyte-depleting conditioning regimens commonly employed in such therapeutic strategies.

**Table 2:** Human papilloma virus (HPV)-specific adoptive cell therapies (ACTs)

ACTs	Related cancer	Biological effect	Limitations	Combinations	Adverse events	Tumor responses	Trial setting	Ref.
E7_11 − 19_ TCR-T	Metastatic HPV16-positive epithelial cancer cells	Extensive regression of bulky tumors and complete regression of most tumors	HLA-A*02:01 restricted; Tumor-intrinsic resistance mechanisms: antigen presentation and interferon response defects	Lymphocyte-depleting chemotherapy and aldesleukin	Grade 3/4 from conditioning regimen and high-dose aldesleukin	6/12 ORR	Phase I NCT02858310	[31]
E6_92 − 101_TCR-T	CC	Antigen-specific activation in vitro and tumor inhibition in vivo	HLA-A*11:01 restricted; Not fully eradicate tumors.	Intraperitoneally inject IL-2	Not mentioned	Efficiently repress tumor growth	Murine model	[32]
E6_29 − 38_TCR-T	Metastatic HPV16-positive epithelial cancers	Partial regression	HLA-A*02:01 restricted; Treatment resistance due to genetic defects in IFNγ response or antigen presentation, PD1 expression	Lymphocyte-depleting chemotherapy and aldesleukin;	No autoimmune adverse events or off-target toxicities, no acute toxicities or cytokine storm; Grade 3/4 adverse events	2/12 ORR	Phase I/II NCT02280811	[34]
HPV-TILs	Metastatic CC	Reactivity against HPV-16 or HPV-18 E6 and E7; Durable, complete or partial regression		Lymphocyte-depleting chemotherapy and aldesleukin	No acute toxicities, no autoimmune adverse events; Grade 3 and 4 adverse events: infection complex, hematologic and the expected result of the lymphocyte-depleting conditioning regimen	3/9 ORR, 2/9 CR	Phase I/II NCT01585428	[38]
HPV-TILs	HPV-associated epithelial cancer cells	Durable, complete or partial regression	HPV-reactive T cells are a relatively small fraction of the infused TILs	Lymphocyte-depleting chemotherapy and aldesleukin;	No acute infusion-related toxicities and no autoimmune adverse events, expected hematological toxicities of the conditioning regimen	2/29 CR, 5/18 ORR in CC 2/11 in noncervical cancer	Phase II	[39]
E7-DCs	stage IV CC II	Inducing specific immune responses in vivo, well-tolerated and no toxicity	No histopathological regression and viral clearance, severe or complete loss of HLA expression	Recombinant HPV16/18 E7	Well-tolerated and no local or systemic side effects or toxicity	No ORR, antibody response in 3/11, cellular immune response in 4/11 patients	Phase I	[51]

### HPV-specific CAR-T cells

The E7 oncoprotein localizes to the intracellular compartment and consequently cannot be targeted with antibodies or generic CAR-T cells. In the HPV-infected epithelium, E7 proteins are cleaved into short peptide fragments by proteasomes and presented on the cell surface by HLAs, and thus recognized and attacked by T-cells. We presume that CAR-T cells targeting antigen-peptide HLA complexes have higher specificity and lower off-target toxicity. Novelly constructed CAR-T cells targeting the E7_11 − 19_/HLA-A^*^02:01 complex (E7_11 − 19_:HLA CAR-T) are waited for intensively investigated. Upon delivery using viral vectors, extracellular vesicles or some targeting sequences, the intracellular single-chain antibodies (scFvs) counteracting the E6 and E7 oncoproteins demonstrated antitumor efficacy in vitro and in vivo [26–28]. To our knowledge, CAR-T cells targeting the oncovirus-restricted surface antigen have not yet been reported.

### HPV-specific TCR-T cells

TCR-T cells that recognize both surface and internal viral antigens demonstrate robust infiltration and persistence in the treatment of solid tumors. As a model for proof-of-principle studies in epithelial cancers, the treatment of HPV-induced cancers using virus-specific TCR-T cells has been established [29]. Epitope E7_11 − 19_-targeted and HLA-A^*^02:01-restricted TCR transgenic T (E7_11 − 19_ TCR-T) cells can specifically recognize and kill HPV16-positive cancerous cell lines and mediate regression of established human HPV16-positive CC in an in vivo model [30]. These data provide preclinical support for first-in-human, phase I clinical trials of such E7_11 − 19_ TCR-T cells. Robust tumor regression was demonstrated as clinical objective response rate (ORR) in 6/12 metastatic HPV-associated epithelial cancers, including complete remission (CR) of lesions and marked responses even in anti-PD-1 refractory patients [31].

TCR-T cells directed against E6_92 − 101_ of HPV16 (E6_92 − 101_TCR-T) were validated high activity towards HPV16-positive HLA-A^*^11:01 CC cells in vitro and efficiently repressed tumor growth in a murine model [32]. Another earlier study revealed that the avid E6_29 − 38_TCR-T, recognized an HLA-A^*^02:01-restricted epitope of HPV16 E6 successfully targeted HPV16-positive epithelial tumor cells and caused tumor regression [33]. In a subsequent phase I/II study, E6_29 − 38_TCR-T cells showed high levels of peripheral blood engraftment 1 month after treatment and induced an ORR in 2/12 patients with chemotherapy-refractory, metastatic HPV16-positive epithelial cancers [34]. Overall, TCR-T therapy based on E6 and E7 oncoproteins of HPV16-positive epithelial cancers are capable of in vivo expansion, long-term persistence, and tumor regression.

### HPV-specific TILs

Therapeutic TILs for successful treatment of patients with HPV-positive CC are directed for HPV E6 and/or E7 antigens [35–37]. However, that bulk of E6 and E7 specific T-cells reside predominantly in the PD-1-expressing T-cell compartment, are rendered functionally inactive within the TME and display no preferential in vivo expansion [35, 36], suggesting that PD-1 blockade or proper stimulation can be exploited for unleashing diverse antitumor T-cell reactivities. Using a lymphocyte depletion chemotherapy regimen followed by aldesleukin administration and TIL infusion, 9 women with metastatic CC participated in the reported phase I/II clinical trial of single infusion of E6 and E7 targeted TILs (HPV-TILs). Ultimately, 3/9 patients experienced objective tumor responses, whereas 2/9 patients experienced CR 15 and 22 months after treatment [38]. In the subsequent phase II study, 5/18 patients with metastatic CC showed ORR, 2 of whom achieved CR, and 2/11 HPV-positive noncervical cancer cohort also showed ORR. Even more importantly, the magnitude of HPV reactivity and peripheral blood repopulation with HPV-TILs are correlated with the clinical response [39]. These studies indicate that HPV-TIL therapy is feasible and can induce persistent CR in metastatic HPV-positive CC.

### HPV-specific DC vaccines

DCs orchestrate adaptive immunity by phagocytosing viral antigens and presenting peptide epitopes, but only a limited number of peptide epitopes are capable of priming specific CTL precursors for a given HLA. More concerningly, the inadequate antigen presentation on mature DCs is frequently observed in HPV-positive tumor bearing individuals [40–42]. Although cognate peptide-loaded autologous DCs can stimulate a specific CTL response against HPV16 E7_86 − 93_, such immunogenic peptide does not appear to be processed or presented by HPV16-infected cells [43]. This finding raises controversy regarding the antitumor activity of HPV-specific CTLs and challenges our understanding of CTL epitope mapping.

To broaden clinical applicability, diverse strategies employing genetically modified DCs expressing E6/E7, pulsed with E6/E7 fusion proteins, infected with recombinant adenovirus, or hybridization with tumor cells have facilitated a versatile presentation of all possible CTL epitopes [43–45]. To this end, DC-based vaccines can overcome the limitations of peptide epitopes with respect to specific HLA-haplotypes and improve antigen presentation in tumor-bearing individuals. As expected, intramuscular administration of E7-transfected murine DCs substantially decreased tumorigenicity and generated strong immunity against HPV16/18 E7-expressing neoplasms [46]. The enhancement of immune responses by cytokines and immunostimulatory gene therapy should prospectively potentiates antitumor activity, as extensively reviewed elsewhere [44, 47, 48]. However, the immune inhibitory effect on HPV16 E7-expressing DCs has been shown to be mediated by the coexpression of IL12 [49]. In preclinical models, local administration of the tumor lysate-pretreated DC vaccines, containing HPV16 E6/E7 oncoprotein, effectively inhibited recurrence or minimal residual tumors in mice [50].

In a pilot clinical study of 15 CC patients, HPV E7 antigen-loaded autologous DCs (E7-DCs) induced a specific antibody response in 3/11 evaluated patients and a specific cellular immune response in 4/11 patients. Unfortunately, neither histopathological regression nor viral clearance of treated patients was observed, attributed to HLA expression loss [51]. Moreover, even with a sharp decrease in L1 expression and a limited cellular immune response against the L1 antigen in cervical lesions with established HPV16 infection, DCs pulsed with major L1 capsid protein-based HPV16-like particles (VLPs, L1-DCs) can elicit strong specific CTLs and lyse HPV16-infected autologous tumor cells [52]. Evidence also suggests that CTLs induced by VLP vaccination can target cells expressing low L1 protein levels [53, 54]. Collectively, ongoing optimization of DC-based cancer vaccines is essential given their current therapeutic limitations in HPV-related cancers.

## HBV

HBV is a hepatocyte specific and enveloped DNA virus. The HBV genome contains four partially overlapping open reading frames (ORFs), namely the preS/S region, preC/C region, P region, and X region. According to the different starting codon positions, preS/S can be divided into three different structural domains, including preS1/S, preS2/S, and S, which are responsible for the large protein (LHB), medium protein (MHB), and small protein (SHB) of the envelope protein hepatitis B surface antigen (HBsAg), respectively. Upon entry into hepatocytes, the virus releases its relaxed circular DNA (rcDNA), which is then transported to the nucleus where it forms covalently closed circular DNA (cccDNA). Transcription of cccDNA generates pregenomic RNA (pgRNA), which serves as a template for both viral genome replication and the translation of viral proteins. Newly synthesized pgRNA is encapsidated along with the viral polymerase to form nucleocapsids. Within nucleocapsids, the reverse transcription process generates rcDNA, which can either replenish the cccDNA pool or be enveloped and released as mature virions [55]. This replication cycle perpetuates HBV infection and contributes to its persistence in the host. Owing to the reverse transcription process of HBV, its DNA can be integrated into the chromosome of the targeted cell, resulting in genomic instability, direct insertional mutagenesis and abnormal expression of oncogenes and tumor suppressor genes [56].

Persistent HBV infection predisposes to the initiation and development of hepatocellular carcinoma (HCC) through necro-inflammation and direct carcinogenic effects. The prevalent HBV-related HCC (HBV-HCC) is mostly characterised by HBV-DNA integrations, even in cases serologically negative for HBV antigens [57]. Additionally, the immunosuppressive TME facilitates virus escape and chronic HBV (CHB) progression. The oncogenic process is multifaceted, involving intervention in various signal pathways through microRNAs, compromised immune responses, increased chromosomal alterations, endoplasmic reticulum (ER)-stress toward hepatocellular transformation, epigenetic dysregulation of tumor suppressor genes, and overexpression of fetal liver/hepatic progenitor cell genes [58, 59]. In terms of a plethora of oncogenic factors, prolonged expression of the viral HBV x antigen (HBxAg) and aberrant preS1/S2 envelope proteins dysregulate cell transcription and proliferation, making liver cells sensitive to carcinogenic factors [60–62].

Currently available therapies, including prophylactic vaccine and antiviral treatment, effectively control HBV infection or replication but do not achieve clearance for intermediate and advanced HCC. Functional cure, defined by HBsAg loss, does not equate to viral eradication in CHB patients, as residual cccDNA or HBV-DNA integrations encoding HBsAg can lead to disease relapse in HBV carriers [63]. Purging cccDNA in hepatocytes through deamination-induced decay following antiviral therapy is a major therapeutic goal in CHB [55]. ACTs remain pivotal in the management of infection and in the prevention of HBV-HCC relapse (Fig. 2b; Table 3), although exploitation of HBV antigens as tumor-specific targets for ACTs has been criticized due to their inconsistent expression in HCC. Additionally, risk assessment must address two considerations: the adequacy of cellular immunity to achieve durative and complete HBV clearance in CHB patients subjected to prolonged exposure to HBV antigens; assessing whether the robust cytotoxic effects of ACTs might precipitate severe hepatotoxicity and acute liver damage.

**Table 3:** Hepatitis B virus (HBV)-specific adoptive cell therapies (ACTs)

ACTs	Biological effect	Carcinogenic traits	Limitations	Combinations	Adverse events	Tumor responses	Trial setting	Ref.
HBsAg-CAR-T	Recognize all the extracellular and secreted HBsAg, recognize different HBV subtypes, engraft and expand in vivo	Lacking cccDNA formation	Short HBsAg-CAR-T cell persistence and the rising viral parameters	Lymphodepletion before cell transfer is not necessary	Transient liver damage	Reduce HBV replication	HBV-transgenic mouse model	[66]
SHB-CAR-T	Induce long persistence and trigger antibody responses, show sustained antiviral effect	AAV-HBV infection	Un-cure the HBV infected cells or HBV-induced HCC	Irradiation and tolerization of immunocompetent mice, lymphodepleting regimens are contraindicated	Not mentioned	Reduce HBV replication	Immunocompetent mice	[67]
HBsAg-CAR-T	Accumulate within the liver, decrease in plasma HBsAg and HBV-DNA levels	Harboring HBV cccDNA and mimicking HBV infection	Noncytopathic killing		Not mentioned	Induce viral clearance	HBV-infected humanized liver chimeric mice	[68]
preS1-CAR-T	Induce much more apoptosis in HCC organoids, increase secretion of IFNγ and stronger antitumor effect in a PDX model, exhibit tumor-reactive marker CD39^+^	HBV-HCC		Triple knockdown of exhaustion markers (PD-1, Tim-3, and Lag-3)	Not observed	Induce apoptosis in HCC, potently inhibit tumor growth	HCC organoids and PDX mouse model.	[69]
HBsAg- and HBcAg-TCR-T	Eliminate HBV infection and suppressed virological markers	HBV-HCC, CHB	HLA-A*02 restricted	Co-treatment with the entry inhibitor Myrcludex B ensured long-term control of HBV infection	Without damaging non-infected cells	Specifically clear HBV infected cells	HBV-infected, humanized mice	[73]
HBsAg- and HBcAg-TCR-T	Lower levels of perforin and granzyme, nonlytic HBV inhibition	HBV-HCC, CHB	HLA restricted	Electroporate resting T cells with mRNAs	Without lysing HBV infected cells	Limit HBV infection	HBV-infected, humanized immunodeficient mice	[74]
HBsAg_183 − 191_-TCR-T	Expand and HBsAg drop without exacerbation of liver inflammation or other toxicity	HBV-HCC metastasis with HBV-DNA integration after a liver transplant	HLA-A*02:01 restricted, no clinical efficacy	Liver transplant, anti-HBV drugs, no pre-emptive lymphodepletion or cytokine therapy	Without exacerbation of liver inflammation, no on-target liver toxicity or other toxicity	Reduce the volume of the HCC metastasis	First-in-man proof-of-concept clinical trials	[75, 77]
HBsAg_183 − 191_-TCR-T HBsAg_171_-TCR-T	Reduce or stabilize circulating HBsAg and HBV DNA levels	Advanced HBV-HCC patients	Adverse events 2/8, HLA-A*02:01, HLA-C*08:01	mRNA electroporating, liver transplantation	Grade 3 liver-related adverse	1/8 patients achieve PR and last for 27.7 months.	Phase I NCT03899415	[78]
HBsAg or HBcAg-TCR-T	Eliminate HCC cells expressing HBV antigens in vitro and in vivo, transient self-limiting inflammatory reaction and no integration into the host genome,	HBV-HCC recurrence post-liver transplant	HLA-A*02:01-HBsAg, HLA-A*11:01-HBcAg, HLA-B*58:01-HBsAg or HLA-C*08:01-HBsAg restricted	mRNA electroporating, liver transplantation	Grade 1 pyrexia, no cytokine release syndrome nor neurotoxicity	No superior anti-tumor efficacy, 1/6 ORR	Phase I NCT02719782	[80]
HBsAg_344 − 377_-TCR-T	Recognize and activate by short epitopes encoded by integrated HBV-DNA	HBV serologically negative HCC relapses with HBV-DNA integrations after liver transplantation	HLA-B*5801 restricted	mRNA electroporating, liver transplantation	No notable adverse events	Decrease volume of pulmonary metastases	Proof-of-concept study	[72]
HBsAg_183 − 191_-TCR-T HBsAg_171_-TCR-T	Transient immunological alterations	CHB and diffuse nonoperable HBV-HCC	HLA-A*02:01, HLA-C*08:01 restricted	mRNA electroporating, Sorafenib treatment	Well tolerated with no severe systemic inflammatory events, cytokine storm, or neurotoxicity observed	Destruct tumor lesion or a prolong stable disease in 3/8 patients	Phase I NCT03899415	[81]
HBcAg_18 − 27_ and HBsAg_335 − 343−_loaded DCs	Induce HBV-specific T cells, specifically lyse the transfected hepatocytes and reduce the systemic viral load	CHB	HLA-A*02:01 restricted	HBeAg status determines the stimulation	Not mentioned	Specifically lyse HBV-transfected hepatocytes and reduce the systemic viral load	Immunodeficient humanized mice	[83]
HBsAg/HBcAg-pulsed DCs	Induce strong DC maturation, cytokine production, and enhance capacity to activate antigen-specific CTLs in vitro or in vivo	CHB			Not mentioned	Specific cytotoxicity, immune modulation capacity in some patients	Preclinical	[84–86]
DC-TC	No increase in hepatic transaminases, hepatitis B antigens, or viral DNA	CHB-HCC		Trans-arterial chemoembolization therapy	Not observed	No exacerbating HBV in HCC patients	Phase I	[89]

### HBV-specific CAR-T cells

HBsAg remains positive in both CHB and HBV-HCC with integrated viral genomes. Targeting HBV surface proteins therefore seems promising. SHB and LHB specific CAR-T cells demonstrated recognition of soluble HBsAg and HBsAg-positive hepatocytes, eliciting secretion of IFNγ and IL-2 and selective eliminating of cccDNA-positive cells. More abundance of SHB on the surface of HBV-induced cancer facilitates the ER membrane targeting of HBsAg and steady ER-plasma membrane exchange [14, 64]. Concordantly, SHB-CAR-T cells exhibited faster activation and greater cytokine secretion than LHB-CAR-T cells [65].

In HBV-transgenic mice lacking cccDNA formation, but possessing a functional immune system, large amounts of circulating viral antigens do not inactivate transferred HBsAg-CAR-T cells or lead to uncontrolled immune-mediated damage in vivo. But rather HBsAg-CAR-T cells would recognize all the extracellular and secreted HBsAg proteins (SHB, MHB, and LHB proteins, combined as HBsAg) and demonstrate efficacy against HBV-infected hepatocytes [66]. Reduced HBsAg-CAR-T cell persistence alongside increased viral parameters were observed after initial and sequential transfer into HBV-transgenic mice, suggesting potential causes, such as T-cell exhaustion or overactivation via antigen binding or Fc receptor interaction with the CAR [66, 67]. Notably, through irradiation and tolerization of immunocompetent mice, fully human SHB-CAR-T cell transfer persisted at high numbers and induced a sustained antiviral effect [67]. Accordingly, interactions with the different arms of the endogenous immune system, bystander immune cell activation, and combination therapies are warranted for combating virally induced HCC.

Further preclinical exploration of CAR-T cells as HBV immunotherapy in models with authentic infections harboring episomal HBV cccDNA is warranted. Murine HBsAg-CAR-T cells transferred into HBV-infected humanized liver chimeric mice accumulate in the liver, significantly reducing plasma HBsAg and HBV-DNA levels compared with those in controls [68]. Notably, HBsAg-CAR-T cells did not kill HBV-positive cell lines in cytotoxicity assays, indicating noncytopathic viral clearance. Upon triple knockdown of exhaustion markers (PD-1, Tim-3, and Lag-3), CAR-T cells, which target the preS1 domain of HBsAg and exhibit the tumor-reactive marker CD39^+^ (preS1-CAR-T), potently inhibit tumor growth and increase IFNγ secretion in a patient-derived xenograft (PDX) mouse model [69].

### HBV specific TCR-T cells

HBV-specific TCR-T cells utilize TCR sequences sourced from endogenous T-cells of patients with self-limited HBV infection or are exogenously engineered to recognize HBV antigens presented by infected cells [70, 71]. In some cases, HBV-HCC negative for HBV antigens may contain translationally active HBV-DNA integrations, generating functional T-cell epitopes recognized by and activate HBV-specific T-cells [72].

High-affinity HBsAg- and HBV core antigen (HBcAg)-specific TCRs in resting and activated T-cells from healthy donors and CHB patients can transform these cells into polyfunctional effector cells, which exhibit antiviral efficacy with limited liver injury through direct cytotoxicity [73]. Whilst a single transfer of TCR-T cells into HBV-infected, humanized mice eliminated HBV infection and suppressed virological markers without damaging non-infected cells [73]. Additionally, TCR-T therapy has shown promising results in maintaining memory T-cell function, which is crucial for long-term immune surveillance against HBV reactivation and the occurrence of HBV-HCC. Intriguing, resting T-cells reprogrammed by HBV-specific TCR reduced HBV replication in humanized immunodeficient mice without lysing HBV-infected hepatoma cells and simultaneously have comparable IFNγ levels and lower perforin and granzyme levels [74].

In the first-in-man proof-of-concept clinical trials of TCR-T cells, the HCC-specific antigen HBsAg was targeted by adoptively transferred HBV-specific TCR-T cells in a compassionate setting for HBV-HCC patients with extrahepatic metastasis after liver transplant. Retrovirally transduced TCR-T cells (HBsAg_183 − 191_-TCR-T), which are designed to target HLA-A^*^02:01/HBsAg_183 − 191_ complexes, dramatically reduced HBsAg levels by approximately 10-fold in concomitance with TCR-T cell expansion, albeit with limited survival due to metastatic disease progression [75–77]. These findings underscore the potential efficacy of TCR-T cells targeting HBV antigens in inducing sustained immune control over HBV-related tumors.

In both preclinical and clinical settings, multiple infusions of short-lived mRNA-based HBV-specific TCR-T (HBV-TCR-T) cells for HBV-HCC individuals exhibited clinically relevant suppression of HCC and a reduction or stabilization of circulating HBsAg and HBV DNA levels, indicating on-target effects [72, 78–81]. The transcribed mRNA can be intuitively safe because of the transient self-limiting inflammatory reaction and a dearth of transgene integration into the host genome. However, the results from a phase I trial in a compassionate setting for patients with HCC recurrence post-liver transplant revealed that HBsAg- or HBcAg-directed TCR-T cells engineered by concomitant electroporation of mRNAs encoding specific TCRs have no superior anti-tumor efficacy [80]. From our perspective, the aforementioned status has implications for armoring more robust and drug-resistant TCR-T cells to overcome the immunosuppressive TME.

### HBV-specific DC vaccines

DC-based vaccines loaded with HBV-specific antigens represent a promising immunotherapeutic strategy to restore antiviral immunity crucial for controlling CHB and HBV-HCC [82]. DCs loaded with HLA-restricted peptides such as HBcAg_18 − 27_ and HBsAg_335 − 343_ have demonstrated efficacy in priming specific CTLs ex vivo and in humanized mice. Stimulation of PBMCs or TILs from CHB patients with these peptide-loaded DCs resulted in significant HBV-specific CTL responses, including IFNγ secretion, CD107 expression upon restimulation, reduction in systemic viral load, and lysis of HBV antigen-expressing hepatocytes [83].

Elegant work has validated the safety and efficacy of antigen-pulsed DCs in a large cohort of CHB patients. DCs derived from CHB patients and pulsed with HBsAg or HBcAg effectively induced CTL responses, reversed immune tolerance in CHB, promoted DC maturation, cytokine production, and enhanced CTL activation [84–86]. Since CD14-HBsAg complexes were detected in vitro and in the serum of HBV infected patients. It’s proposed that HBsAg activates DCs through CD14-dependent mechanisms [87], crucial for initiating effective HBV-specific immune responses.

Moreover, DCs loaded with HBV subviral particles (HBVsvp) offer an innovative approach to activate HBV-specific CTLs, bypassing dysfunctional DCs and T-cells in CHB patients, thereby inducing Th1 polarization and strong cytolytic activity [88]. Phase I trials utilizing autologous DCs pulsed with irradiated tumor stem cells (DC-TC) have shown initial safety in patients with cirrhosis and HBV infection, suggesting potential therapeutic benefits [89]. Collectively, these findings underscore the promise of DC-based vaccines as a therapeutic avenue against HBV-associated HCC.

## HCV

HCV belongs to the Flaviviridae family and has a single positive-sense RNA (+ ssRNA), which codifies for an icosahedral nucleocapsid composed of C protein and envelope glycoproteins (E1 and E2), as well as non-structural proteins (NS1, NS2, NS3, NS4A/4B, NS5A/NS5B). The molecular mechanisms underlying HCV-HCC primarily revolves around a complex interplay of viral proteins with cellular pathways, leading to dysregulated cellular functions, genomic instability, and tumor transformation [90, 91].

HCV reaches its peak titers several weeks before the onset of detectable humoral or cellular immune responses and the initiation of liver disease. In western countries and Japan, chronic HCV infection is the primary cause of HCC. Wherein the highly variable HCV genomes under the selective pressure of host immune response are major risk factors for HCC development and impede the effectiveness of prophylactic and therapeutic treatments [92–94]. With the advent of potent antivirals targeting the viral life cycle: the NS3/4A protease, the NS5A protein and the RNA-dependent RNA polymerase NS5B protein, the incidence of HCV-HCC has substantially decreased [95]. Nevertheless, in cases where the HCV titer remains relatively low during chronic infections, we should armor immune cells and pre-empt T-cell exhaustion or anergy to clear HCV infection. HCV specific ACTs are list in Table 4 (Fig. 2c).

**Table 4:** Hepatitis C virus (HCV)-specific adoptive cell therapies (ACTs)

ACTs	Biological effect	Limitations	Combinations	Adverse events	Tumor/virus responses	Trial setting	Ref.
HCV-E2-CAR-T	Specific antigen recognition, degranulation and secretion of proinflammatory and antiviral cytokines	Variable E2, circulating viral particles could interfere or block CAR activity		Induce extrahepatic activation	Clear HCV-infected cells	Proof-of-concept study	[97]
HCV-NS3-CAR-T	Anti-tumor potency via protease-regulated CAR circuits, show diminished T cell exhaustion and greater stemness, enhance anti-tumor efficacy in solid tumor models		HCV-NS3 inhibitor	Control CAR-T cytotoxicity	Reduce tumor and inhibit HCV replication	Proof-of-concept study	[100, 101]
NS3_1073 − 1081_-TCR-T	Induce apoptotic signaling pathways and cause hepatotoxicity	HLA-A2 restricted	In vitro-transcribed TCR mRNA	Hepatotoxic potential	Antigen-specific cytolysis of target cells,	Proof-of-concept study	[104]
NS5_1992 − 2000_-TCR-T	Extend proliferative and metabolic pathways	HLA-A2 restricted	In vitro-transcribed TCR mRNA	No hepatotoxic potential	Non-cytotoxic tumor killing	Proof-of-concept study	[104]
HCV-E2-DCs	Facilitate specific cellular immune activation and induce anti-viral cytokines and antibodies	HLA restricted	HCV peptides loaded-DCs	Not mentioned	Clear virus	Proof-of-concept study	[106]
HCV-NS3-DCs	Activate T cell, enhance cross-presenting capability		Stably express HCV NS3	Not mentioned	Clear NS3-positive hepatocytes	Proof-of-concept study	[107]

### HCV-specific CAR-T cells

The highly variable HCV E2 glycoprotein (HCV-E2) is a major target of the host immune response. Anti-HCV-E2 CARs were designed based on a previously described broadly cross-reactive and cross-neutralising human monoclonal antibody (mAb), directed against conserved HCV-E2 epitopes [96]. The cytotoxic ability of anti-HCV-E2 CARs-grafted T (HCV-E2-CAR-T) cells was evaluated in vitro against HCV-E2-transfected cells as well as hepatocytes infected with HCV. In a proof-of-concept study, retrovirus-transduced HCV-E2-CAR-T were endowed with specific antigen recognition accompanied by degranulation and secretion of proinflammatory and antiviral cytokines [97].

Introducing the HCV NS3 protease (HCV-NS3) between the scFv and hinge domain allowed for protease-regulated CAR circuits, enabling precise control over CAR-T cell activation during cancer therapy. In the absence of HCV-NS3 inhibitor, NS3 displays the proteolytic process, disrupts the CAR structure and prevents the activation signals. Conversely, administering protease inhibitors inhibited NS3 cleavage, preserving CAR integrity and facilitating T-cell activation [98, 99]. The anti-tumor potency and reversibility of drug-regulated CAR-T cells targeting tumor-associated antigens (TAA) were evaluated in solid and hematological tumors [100, 101]. As such, future investigations may explore CARs targeting HCV antigens, potentially leveraging clinically approved HCV-NS3 antiviral protease inhibitors to synergistically combat HCV-HCC.

### HCV-specific TCR-T cells

Two specific HCV TCRs that mounted a polyfunctional response to the cognate HLA-A2-restricted NS3_1073 − 1081_ and NS5_1992 − 2000_ peptide, and enabled to eliminate human hepatoma cells with persistent HCV RNA replication [102]. The expanded study revealed that NS3-specific TCR-T cells were prone to induce the antigen-specific cytolysis of target cells, while NS5-specific TCR-T cells favored a non-cytotoxic mechanism [103, 104], mirroring some marked differences in avidity and functional profile between HCV-specific TCR-T in tumor cell lines. High-avidity NS3-specific TCR-T cells rapidly activated apoptotic signaling pathways, causing hepatotoxicity, whereas the low-avidity NS5-specific TCR-T cells promoted the proliferative and metabolic pathways as the extended survival of HCV target cells [104]. At this juncture we surmised that, high-avidity TCR-T cells demonstrate superior antiviral activity, while low-affinity TCR-T cells are considered more suitable for chronic viral infections due to less immune pathology.

### HCV-specific DC vaccines

Considering the modest immunogenicity of HCV-E2 glycoprotein, modification of HCV-E2 is warranted to applicably potentiate DC function and elicit a robust protective immune response [105]. Moreover, the effectiveness of the DC vaccines loaded with two selected HCV-E2 peptides have been validated to activate peptide-specific cellular immune activation and induce significant levels of anti-viral cytokines and antibodies [106]. As a proof of concept, vaccination with HCV-NS3-expressing DCs (NS3-DCs) in mice played a predisposing role in T-cell activation, cross-presenting capability of DCs in the draining lymph nodes, and clearance of NS3-positive hepatocytes from the livers [107]. DC vaccines, particularly when reinforced by interactions with other immune cells, hold promise for enhancing protective immunity against HCV [108].

## Human CMV

The prevalent human CMV, also known as human herpesvirus 5 (HHV-5), is characterized by a double-stranded linear DNA genome of approximately 235 kb encoding over 200 genes and is implicated in various exoderm-derived malignancies [109–111]. A previous consensus has been reached on the coexistence of CMV and immunocompromised hosts, but the mechanism has not been fully elucidated [112]. More concerningly, CMV can increase cellular proliferation, angiogenesis, and immune evasion, thus enabling several hallmarks of cancer. Intriguingly, anti-CMV VST cells accumulate in extremely high numbers and serve as “bystanders” of the tumor. To harness CMV-specific immunity against malignancies, diverse strategies have been devised to redirect VSTs towards eradicating cancerous cells (Fig. 2d; Table 5) [113].

**Table 5:** Cytomegalovirus (CMV)-specific adoptive cell therapies (ACTs)

ACTs	Related cancer	Biological effect	Limitations	Combinations	Adverse events	Tumor responses	Trial setting	Ref.
CMV-CD19-CAR-T	Intermediate/high-grade B-NHL	Prevent CMV infection or reactivation, anti-CD19 effector functions		Hematopoietic cell transplantation (HCT), CMVpp65 peptide vaccine, CMV-MVA triplex vaccinations	Without graft-versus-host disease, no severe adverse events or dose-limiting toxicities at all dose levels	Median OS of 50.5% at 24 months	Phase I/II NCT01475058	[115]
CMV-Her2-CAR-T	GBM	Exert anti-glioblastoma activity, present in the peripheral blood for up to 1 year, expand at glioblastoma sites	No expansion in the peripheral blood	Without prior lymphodepletion, surgical resections followed by radiotherapy with concomitant temozolomide	Grade 2/3/4 adverse events, well tolerated, with no dose-limiting toxic effects	Median OS of 11.1 months from the first T-cell infusion and 24.5 months from diagnosis, 8/17 PR,	Phase I/II NCT02661282 NCT01109095	[119]
CMV-TCR-T	Recurrent GBM	Exert anti-glioblastoma activity	No significant changes in the polyfunctionality of CMV VST, HLA-restricted	Standard chemotherapy	Well tolerated, and only minor adverse events, grade 1/2/3	Median OS of 4.4–13.4 months and a median PFS of approximately 8.1 months	Phase I ACTRN12609000338268	[121]
CMV-TCR-T	Hematologic malignancies with CMV reactivation after haploidentical peripheral blood stem cell transplants (PBSCT)	Recognize HLA-restricted peptides derived from the CMV pp65, display robust expansion, persistence and rapid CMV clearance in vivo	HLA-restricted	Salvage ganciclovir and foscarnet administration, PBSCT	No severe adverse effects, no immune effector cell-associated neurotoxicity syndrome or TCR-T cell-related GvHD, 1/6 grade 1 CRS, 2/6 mild fever	4/6 ORR without any antiviral agents 2/6 ORR with salvage ganciclovir and foscarnet, median time of CMV clearance is 20.5 days	Phase I NCT05140187	[120]
HA-CMV-TCR-T	AML allogeneic after stem cell transplantation (alloSCT)	Expand and persist, recognize HLA-A*02:01 positive, HA-positive primary AML cells	Predominantly express the triggered TCR, HLA-A*02:01 restricted	without pre-conditioning chemotherapy, alloSCT	No infusion-related toxicity, delayed toxicity or GVHD occurred	3/5 patients were in remission	Phase I EudraCT number 2010-024625-20	[124]
CMV-IE-DCs	Glioma	Induce T cell activation so as to kill cancerous cells and show a prolonged survival in CMV-IE-implanted murine glioma models		Optimized adenovirus targeting the DC surface receptor, DEC205	Not observed	Completely reject cancerous cells when rechallenged	Murine glioma models	[126]
CMV pp65 RNA-loaded-DCs	GBM	Augment polyfunctionality and frequencies of CMV pp65-specific T cells		pp65 mRNA transfection, leukapheresis, chemoradiation, temozolomide	Minor adverse events, no severe adverse events	Improve survival in patients, clinical outcomes of PFS and OS remain unknown	Phase I NCT00693095	[116]
CMV-pp65-DCs	GBM	DC migration to draining lymph nodes		Resection and chemoradiation	Not mentioned	1/3 OS without tumor recurrence at 5 years	Phase II NCT02366728 NCT00639639	[127]

### CAR-redirected CMV-specific cytotoxic T-cells (CMV-CAR-T)

The immunodominant CMV antigens, namely the pp65, IE1, and IE2 proteins, evoke physiological CMV-specific T-cells [114]. These T-cells can be isolated and/or reinvigorated using ex vivo CMV-peptide stimulation prior to CAR transduction, followed by in vivo expansion through a CMV vaccine boost [115–117]. CD19-CAR-T cell therapy faces limitations such as inadequate engraftment, differentiation, exhaustion, prolonged B cell aplasia, and increased susceptibility to CMV infections [118]. In contrast, CMV-CD19-CAR-T cells integrate anti-CD19 effector functions with potent anti-CMV activity, exhibiting superior proliferation, survival, and in vivo antitumor efficacy compared to conventional CD19-CAR-T cells [115]. Moreover, in a phase I dose-escalation trial of progressive glioblastoma (GBM) patients without prior lymphodepletion, Ahmed et al. evaluated the feasibility and safety of CMV-Her2-CAR-T cells and reported a promising median overall survival (OS) of 11.1 months from the first T-cell infusion and 24.5 months from diagnosis [119]. Overall, compared to generic CAR-T cells, CMV-CAR-T cells have shown superior proliferation, survival, and in vivo antitumor efficacy. They are well tolerated with only minor adverse events (Table 5).

### TCR-engineered CMV-specific cytotoxic T-cells (CMV-TCR-T)

To prevent CMV reactivation after haploidentical peripheral blood SCT (PBSCT), a phase I clinical trial assessed the safety and efficacy of CMV-specific T-cells engineered to recognize HLA-restricted peptides from the CMV pp65 protein [120]. Another clinical trial investigated ex vivo expanded CMV-specific T-cells in recurrent GBM patients and reported a median OS of 4.4–13.4 months and a median progression-free survival (PFS) of approximately 8.1 months. However, in vitro analysis did not reveal significant changes in CMV-specific T-cell polyfunctionality [121].

Manufacturing CMV-TCR-T cells appears to be more challenging than CMV-CAR-T cells due to the downregulation of endogenous TCR expression upon forced expression of the artificial TCR. Redirected by the minor histocompatibility antigens (HA), HA-TCR-transferred CMV-specific T (HA-CMV-TCR-T) cells exerted dually potent antileukemic as well as anti-CMV reactivity, showing comparable TCR-specific cytolytic activity to generic TCR-engineered T-cells [122]. Notwithstanding, a follow-up study disclosed that repetitive stimulation skews CMV-TCR-T cells to predominantly express the triggered TCR [123]. In a phase I clinical trial, CMV-TCR-T cells were safely infused into 5/9 patients, but the overall efficacy of this treatment approach was too low to warrant further clinical development [124].

### CMV-specific CAR-T cells

At the early stage of the CMV replication cycle, CAR-T (gB-CAR-T) cells directed against glycoprotein B (gB) accessible on the surface of infected cells can mediate antiviral effector functions, such as cytokine production and cytolysis [125]. However, a set of viral antiapoptotic factors directly abrogate T-cell cytotoxicity at later stages of the replication cycle. These gB-CAR-T cells were not tested in vivo because of the low degree of sequence similarity of gB protein between murine and human CMV, thus, recombinant mouse CMV expressing human CMV-gB is obligatory.

### CMV-specific DC vaccines

A multiperformance recombinant adenovirus coexpressing the CMV immediate early gene (CMV-IE) enables the selective infection of DCs in vivo (CMV-IE-DCs), which induce T-cell activation to kill cancerous cells and prolong survival in CMV-IE-implanted murine glioma models [126]. Owing to the attenuated ability of CMV-specific T-cells in patients to generate multiple cytokines and chemokines, a pilot trial in which 22 patients with GBM received CMV pp65 RNA-loaded-DCs to augment the polyfunctionality of CMV pp65-specific T-cells revealed that polyfunctional T-cell responses are potential biomarkers for effective antitumor immunotherapy [116]. Encouragingly, three separate clinical trials have demonstrated that DC vaccines targeting the CMV pp65 protein (CMV-pp65-DCs) confer long-term survival benefits to nearly 1/3 of GBM patients, showing no tumor recurrence five years posttreatment [127]. Enhanced insights into tumor etiology and immune principles underscore the unique advantages of virus-targeted DC vaccines in specific tumor immunotherapies.

## EBV

EBV belongs to human herpesvirus 4 (HHV-4) of the herpesvirus family and features a 175 kb double-stranded DNA genome encoding over 85 proteins. EBV enters epithelial, B, NK/T cells through a variety of membrane proteins, including gp350, gB, gH, gL and gp42 [128]. EBV has been definitively linked to a variety of lymphoid and epithelial cell malignancies, including B/T/NK cell lymphomas, nasopharyngeal carcinoma (NPC), gastric carcinoma and lung carcinoma due to the immune cell exhaustion and dysregulation [129, 130]. Upon primary infection, the immune-evasive EBV establishes latency and allows the viral genome to persist in the lymphatic system by driving the expansion of infected B cells [131]. EBV-infected B cells selectively express latent viral proteins: EBV nuclear antigen (EBNA) and latent membrane proteins (LMPs). As signaling proteins, LMPs promote the overexpression of some TAAs in B cells and upregulate costimulatory ligands to jointly activate T-cells [132]. As importantly, continuous EBNA1 expression is crucial for maintaining EBV genome replication in EBV-positive tumors, whereas EBNA3 is upregulated in EBV-induced lymphoma and can induce potent anti-EBV-specific CTLs. In addition, compared with those in the control lymphocytes of healthy individuals, EBNA1 and EBNA3 mRNA levels in EBV-induced lymphoma cells are increased by thousands of folds [133]. Accordingly, these latent EBV proteins are viable targets for cellular immunotherapies to clear EBV-infected targets (Fig. 2e; Table 6).

**Table 6:** Epstein-Barr virus (EBV)-specific adoptive cell therapies (ACTs)

ACTs	Related cancer	Biological effect	Limitations	Combinations	Adverse events	Tumor responses	Trial setting	Ref.
LMP/EBNA1-CTL	Recurrent and metastatic NPC	Induce LMP- and EBNA1-specific T cells, control tumor progression	27.3% patients showed minimal or no expansion	Irradiated autologous PBMCs infected with AdE1-LMPpoly	Grade 1/2 toxicities including flu-like symptoms, malaise, dry cough, and low blood pressure, a single case of a serious adverse event (SAE) due to progressive disease	Median OS of 17.2 months	Phase I ACTRN12609000675224	[134]
LMP-CTL	EBV-associated lymphoma	Eradicate lymphoma, observe epitope spread in in CR patients		Autologous DC and B LCLs transfected with adenoviral vectors (Ad5F35) expressing LMP antigen	No infusional toxicities	13/21 ORR, 11/21 CR, 28/29 sustained remission at a median of 3.1 years after CTL infusion	Phase I NCT00671164	[136]
Ad-ΔLMP1-LMP2-DCs	EBV-positive advanced NPC	Activate LMP1/2-specific T cells in vitro	No increase in the frequency of peripheral LMP1/2-specific T cells	Ad-ΔLMP1-LMP2 transduction,	Induced delayed type hypersensitivity responses, no significant toxicity was observed	3/12 ORR, 1/12 PR, 2/12 SD, median PFS was 1.92 months, e median OS time was 6.0 months	Phase II	[140]
LMP2-DCs	NPC	Boost LMP2 specific CTL, control and prevent NPC recurrence and metastasis		Ad-LMP2 transduction	Well tolerated	Five-year OS rate of 94.4% in responders	Phase I	[141]
LMP1-CAR-T	NPC	Be activated in co-culture with NPC cells overexpressing LMP1 and produce IFNɣ and IL-2 in a LMP1 specific manner			Not mentioned	Reduce tumor growth	Xenograft mouse	[142]
gp350-CAR-T	EBV-associated malignancies, lymphoproliferative disease	Exert cytotoxicity and impend EBV-associated lymphoproliferation and lymphomagenesis	Lytic viral protein gp350	Transplant with cord blood CD34^+^ cells and infect with the EBV/M81/fLuc lytic strain	Not mentioned	75% of mice control or reduce EBV spread and showed lower frequencies of B cell malignant lymphoproliferation, lack of tumor development, and reduce inflammation.	Humanized mouse	[143, 144]
off-the-shelf edited gp350-CAR-T	Lymphoma	Exert low efficacy on lymphoma but reduce the EBV DNA load in the bone marrow	Weak and variable expression of gp350, promote gp350 antigen escape	CRISPR/Cas9 gene editing methods to knock in CAR, infect with a lytic type 2 EBV	Not mentioned	Do not inhibit tumor growth in vivo but reduced the EBV DNA load in the bone marrow and promote gp350 antigen escape	Xenograft mouse	[145]
LMP1-TCR	EBV-associated malignancies	High avidity, provoke cytokine secretion and cytolytic activity, ex vivo proliferate	HLA-A*0201 restricted	Transplant with cord blood CD34^+^ cells and infect with the EBV/M81/fLuc lytic strain	Not mentioned	Inhibit tumor growth	Xenogeneic mouse	[146]

### EBV-specific CTLs

EBV-specific CTLs face challenges due to EBV’s variable viral gene expression and multiple evasion mechanisms, complicating epitope selection. During the latent phase, EBV-specific CTLs are often infrequent, relatively immature, and anergic, potentially allowing tumor cells to evade immune surveillance. Previous studies have demonstrated that expanding LMP1/EBNA1-specific CTLs by coculturing with irradiated autologous PBMCs infected with an adenoviral vector encoding EBNA1 and multiple CTL epitopes from LMP1 and LMP2 (AdE1-LMPpoly), followed by reinfusion into EBV-positive recurrent and metastatic NPC patients, effectively controlled tumor progression with a median OS of 17.2 months [134]. However, preparative lymphodepleting chemotherapy prior to administering higher doses of EBV-specific CTLs did not improve clinical outcomes in patients with EBV-associated NPC [135]. In addition, LMP specific CTL was expanded using autologous DCs and EBV-transformed B-lymphoblastoid cell lines transduced with an adenoviral vector expressing LMP, which could induce durable CR in lymphoma patients at a median of 3.1 years after CTL infusion. Within 2 months after CTL infusion, epitope spread can be detected in patients who achieve clinical responses [136]. To enrich BARF1-specific CTLs for NPC treatment, EBV lytic cycle inducers can be used to upregulate the BARF1 oncogene in LCLs to promote more pronounced immunogenic properties [137, 138], suggesting new strategies to bolster EBV-targeting immunotherapy.

Initial preparation of EBV-specific CTLs involved stimulating PBMCs with autologous EBV-transformed LCLs, followed by transduction with E1-deficient adenovirus. These E1-transgenic CTLs released oncolytic adenovirus at tumor sites, leading to tumor regression upon exposure to HLA-matched, EBV-infected cells [139]. Recent research highlights the potential of ectopically expressed LMP1 in tumor B cells to prime autologous CD4^+^ T-cells (LMP1-CD4^+^ T) against a wide array of endogenous tumor antigens, including TAAs and neoantigens, suggesting efficient treatment for B cell malignancies [132]. These groundbreaking studies underscore the necessity of reevaluating conventional paradigms in both viral and tumor immunity.

### EBV-specific DC vaccines

Although autologous DCs transduced with an adenovirus encoding truncated LMP1 (ΔLMP1) and full-length LMP2 (Ad-ΔLMP1-LMP2-DCs) enable to activate LMP1/2-specific T-cells in vitro, no increase in the frequency of peripheral LMP1/2-specific T- cells was detected in advanced NPC patients. Meanwhile, they induced delayed-type hypersensitivity responses but did not result in significant toxicity [140]. Considering its limited efficacy, future research should prioritize the administration of more potent DC vaccines to patients with lower tumor burdens. In a pilot study of 29 subjects, intradermal injection of LMP2-DCs using an adenovirus expressing LMP2 (Ad-LMP2) achieved a five-year survival rate of 94.4% in NPC responders, indicating enhanced responses to LMP2 peptide pools [141].

### EBV specific CAR-T cells

Compared to CTL treatment regimens, the development of EBV-specific CAR-T is somewhat slower. CAR-T cells engineered with the scFv specific to the extracellular domain of LMP1 (LMP1-CAR-T) were activated in co-culture with LMP1-overexpressing NPC cells, leading to production of IFNɣ and IL-2. Intra-tumoral injection of LMP-CAR-T cells in a xenograft mouse model reduced tumor growth [142]. Moreover, a clinical trial is currently underway (NCT02980315) to evaluate LMP1-CAR-T cells for treating EBV-associated malignant tumors.

The lytic envelope gp350 is prominently expressed on the surface of cells during EBV lytic reactivation and persists in subsets of latently infected cells. A proof-of-concept preclinical study revealed that gp350-targeting CAR-T cells (gp350-CAR-T) exerted cytotoxic effects against EBV-positive tumor cells and hindered EBV-associated lymphoproliferation and lymphomagenesis in a fully humanized mouse model [143, 144]. However, using TCR alpha chain (TRAC) locus-knock-in, off-the-shelf edited gp350-CAR-T cells showed limited efficacy against lymphoma due to weak and variable gp350 expression [145], highlighting EBV’s immune evasion mechanisms that can affect CAR-T cell characteristics and efficacy.

### EBV-specific TCR-T cells

The TCR specific to LMP1 (LMP1-TCR) provoked high levels of cytokine secretion and cytolytic activity, displaying explosive ex vivo proliferation upon antigen activation, and inhibited tumor growth in a xenogeneic model [146]. Ongoing efforts aim to generate more robust EBV TCRs by incorporating a CD28 domain preceding CD3, which augments antigen-specific IFNγ production without compromising the cytotoxic response [147]. Clinical trials investigating LMP2-specific TCR-T cells are ongoing (NCT04509726, NCT03925896). Despite promising results, the effectiveness of adoptive immunotherapy for EBV-associated cancers remains constrained by limited targetable EBV antigens and their suboptimal immunogenicity.

## HIV-1

HIV-1 latent reservoirs are established days after infection and persist through clonal expansion of infected cells. Individuals living with HIV-1 face heightened risks of developing T-cell lymphoma and B-cell non-Hodgkin’s lymphoma (B-NHL), predominantly diffuse large B-cell lymphoma (DLBCL) and Burkitt’s lymphoma [148]. Pathogenesis studies highlight multifaceted mechanisms encompassing oncogenic proteins, immune system dysregulation, genetic predisposition, and other factors. Despite antiretroviral therapy (ART) effectively suppressing active viral replication, it fails to eliminate integrated latent viruses, necessitating lifelong treatment. Strategies to target HIV-1 latent reservoirs and associated lymphomas propose cytolytic immunotherapies as adjunctive to ART (Table 7; Fig. 2f).

**Table 7:** Human immunodeficiency virus type 1 (HIV-1)-specific adoptive cell therapies (ACTs)

ACTs	Biological effect	Limitations	Combinations	Adverse events	Tumor/virus responses	Trial setting	Ref.
CD4-CAR-T	Inhibit viral replication, kill HIV-1-infected cells in vitro, and survive for prolonged periods in vivo	Underwent lympha pheresis, CD4-CAR-T cells are susceptible to HIV-1-1 infection	Antiretroviral therapy (ART), Subjects with undetectable plasma viremia	No serious related adverse events, the majority of adverse events related to T cells were mild	Viral burden was not substantially altered in patients, a trend toward fewer patients with recurrent viremia	Phase II	[151]
bNAb-derived CAR-T	Decrease viral RNA and intact proviruses, reduce virus diversity and viral reservoir. safe and well tolerated	Viral escape mutations emerge, viral rebound due to preexisting or emergence of viral escape mutations	Cease ART	No serious adverse events	Reduce viral reservoir	Phase I NCT03240328	[152]
anti-HIV-1 duoCAR-T	Target gp120 and gp41, reduce cellular HIV-1 infection, eliminate PBMCs infected with broadly neutralizing antibody-resistant HIV-1 strains	Confounding effects of a reconstituted endogenous T cell immune response	Donor-matched PBMC are activated with IL-2 and PHA	Not mentioned	Reduce cellular HIV-1 infection by up to 99% in vitro and > 97% in vivo	Humanized NSG mouse model of intrasplenic HIV-1 infection	[154]
anti-HIV-1 duoCAR-T	Target gp120 and gp41, early memory phenotype T cell localize to spleen and eliminate HIV-1-infected PBMCs, kill HIV-1-infected CD4^+^ T cells and monocytes/macrophages		Cease ART	Not mentioned	Eliminate HIV-1-infected PBMCs	Phase I/II NCT04648046	[155]
anti-HIV-1 TCR-T	Show robust, antigen-specific polyfunctional cytokine profiles, allow TCR-T cells to recognize HIV-1 escaped epitopes	Enhanced TCR affinity cannot augment HIV-1 inhibitory, speed of target recognition and killing is lower than CAR-T cells		Not mentioned	Lack HIV-1 suppression	Preclinical	[156]
HIV-1-DC	Control of HIV-1 replication, decrease plasma viral load, increase HIV-1-specific T cell responses		Pulse with heat inactivated autologous HIV-1, ART interruption	Safe and well tolerated	a decrease of plasma viral load setpoint ≥ 1 log in 12/22	NCT00402142	[158]
DC-TRN	Enhance HIV-1-specific T-cell responses	No significant correlation with time off treatment	Electroporate Tat, Rev and Nef encoding mRNA, ART interruption	Safe and well tolerated	No correlation with clinical parameters could be found	Phase I/IIa NTR2198	[159]
DC-TRN	Induce changes in natural killer cell phenotype and functionality		Electroporate Tat, Rev and Nef encoding mRNA, ART interruption	Safe and well tolerated	NK cell-mediated HIV-1 killing	Preclinical	[157]

### HIV-1-specific CAR-T

The huge success of CAR-T therapy for B cell leukemias is rooted in pioneering preclinical and clinical study of HIV-1 infection. Furthermore, CAR-T cell has been recommended for the clinical therapy for HIV-1-positive lymphoma patients [149, 150]. In a randomised phase II clinical trial, first-generation CAR-T cells using CD4 ectodomain (CD4-CAR-T) to target the HIV-1 gp120 expressed on the surface of HIV-1-infected cells, noted a trend toward viral-load rebound and long-term engraftment in patients [151]. Given that CD4 and CCR5 are primary coreceptors of HIV-1 infection, CD4-CAR-T cells are susceptible to HIV-1 infection. For that reason, broadly neutralizing antibodies (bNAbs) against HIV-1 are engineered in the CAR construct (bNAb-derived CAR-T) cells showed a higher neutralizing capacity for different HIV-1 strains and circumvented HIV-1 infection [152]. Furthermore, bNAb-derived CAR-T cells with the deletion of CCR5 exhibited superior viral replication control compared to counterparts lacking this modification [153]. Nevertheless, emergence of resistant viral variants through spontaneous mutations poses a challenge to sustained efficacy, necessitating ongoing refinement. Innovative approaches like DuoCAR-T cells, targeting multiple binding sites on gp120 and the extracellular region of gp41, exhibited promising efficacy in eliminating HIV-1 in preclinical models of humanized mice with intrasplenic infection, presenting a multifaceted strategy against globally prevalent HIV-1 strains [154, 155].

### Other HIV-1-specific ACTs

Anti-HIV-1 TCR-T cells manifested robust, antigen-specific polyfunctional cytokine profiles upon encountering antigens, but ineffectively controlled HIV-1. Conversely, CAR-T cells demonstrated accelerated recognition and elimination of HIV-1-infected targets relative to TCR-T cells, attributed to their ability to activate Caspase 3 and induce apoptosis in HIV-1-infected cells [156]. Therefore, it is hypothesized that the speed of target recognition and killing determines the efficacy of engineered T-cell therapies for infectious HIV-1.

Therapeutic DC-based vaccines pulsed with heat inactivated autologous HIV-1 (HIV-1-DC) have shown feasibility, safety, and well-tolerated outcomes in clinical settings [157]. Additionally, DCs electroporated with mRNA encoding Tat, Rev, and Nef (DC-TRN) significantly modulated NK cell and HIV-1-specific T-cell responses, leading to substantial reductions in plasma HIV-1 viral loads following interruption of antiretroviral therapy [158, 159]. These findings underscore the potential of DC-based approaches in augmenting immune responses crucial for controlling HIV-1 infection and HIV-1-defined cancers.

### Other oncoviruses in viral malignancies

KSHV is etiologically linked to Kaposi’s sarcoma and primary effusion lymphoma, where both latency and lytic reactivation phases contribute to the pathogenesis of KSHV-associated malignancies [10, 160]. Notably, KSHV’s immune evasion strategies, mediated by genes like KSHV K3 and K5 encoding membrane-tethered E3 ubiquitin ligases, interfere with MHC expression, thereby evading immune surveillance by T and NK cells. This evasion mechanism could be exploited in the development of off-the-shelf allogeneic CAR-T cells. Incorporating K3 or K5 into CAR constructs has been shown to decrease the recognition and cytotoxicity against allogeneic T-cells in both culture and animal models [161].

Akin to MCPV, the elevated prevalence and viral load of polyomavirus JC (JCPV) within tumor tissues strongly suggest an active role in tumorigenesis rather than a bystander effect [162–164]. Furthermore, some lymphomas are characterized as virus-associated cancers due to the high incidence of viruses such as HIV-1, EBV, KSHV, HCV, HBV, and others, all of which exert pathogenic effects [90, 165]. Cooperative interactions between different oncoviruses represent an additional contributory mechanism in viral malignancies [165].

## Conclusions and perspective

Preclinical and clinical studies have sought to utilize a flood of innovation ACTs for the prophylaxis and treatment of virus infection in both refractory and advanced malignancies [63, 166–168]. Capitalizing on the etiological link between viral malignancies and oncoviruses, we have summarized the relevant literature on the use of virus-specific ACTs to avoid or ablate viral malignances, and this information may also provide guidance for the selection of effective oncovirus-encoded antigens (Fig. 2a-f). This therapeutic approach is often combined with vaccinations, immune checkpoint inhibitors, systemic aldesleukin, virotherapy, and support by organ transplantation. Notably, virus-specific ACT mediated antitumor effects were observed even in heavily pretreated patients. These immune antitumor effects may be even more clinically evident when used as a first-line treatment at the early stage of virus infection, since an intense immunosuppressive TME that is typically encountered in refractory cancer patients may not be present.

The bottleneck of ACTs for treating viral malignancies lies in several factors, including inadequate expansion and persistence of adoptive cells, MHC downregulation, suppressive TME, and targetable viral antigen level. Traditional strategies for solid tumor treatment aimed at increasing the trafficking, infiltration, and persistence of highly active adoptive cells are also applicable to the treatment of viral malignancies. Specially T-cells can be engineered with costimulatory signaling, immune checkpoint inhibitors, CMV/EBV TCR coexpression, and tissue homing ligands, which have demonstrated several advantages over the prototype, including enhanced expansion, persistence, antiviral activities. One major challenge is effectiveness of this therapy in recognizing and targeting infected cells. This is largely dependent on the ability of the transferred T-cells to interact with MHC molecules presenting viral antigens on the surface of infected cells. Another crucial issue is the TME within the infected tissue, which can create an inhibitory environment that hinders the function of the transferred cells. This may include factors such as immune suppressive cells, cytokines, and a lack of adequate nutrients for T-cells to proliferate and function optimally. Furthermore, the availability and selection of appropriate viral antigens for targeting also play a significant role in ACT success. In addition, an important direction for future research involves targeting multiple highly conserved sites of more than one viral antigen and utilizing a variety of therapeutic targets to overcome the viral escape mechanisms. Identifying highly immunogenic and conserved antigens that elicit a strong T-cell response is essential for effectively clearing the viral infection. Overall, overcoming these obstacles requires a comprehensive understanding of the immune response to viral infections and the development of strategies to optimize the function of adoptive cells in the context of the complex TME. Furthermore, investigating the optimal timing for intervening in the progression from viral infection to chronic inflammation to cancer development is crucial. Early intervention strategies to prevent or delay the carcinogenic process represent a significant area for further exploration and discussion in the field of cellular immunotherapy for viral infections. In general, ACTs can target viral antigens and tumor-specific markers, and provide potent immune responses against viral infections and their associated malignancies. More importantly, we need to determine which specific scenarios can be administrated by certain form of ACTs.

## Data Availability

No datasets were generated or analysed during the current study.

## Abbreviations

ACTs: Adoptive cell therapies
alloSCT: Allogeneic after stem cell transplantation
ART: Antiretroviral therapy
B-NHL: B-cell non-Hodgkin’s lymphoma
CAR: Chimeric antigen receptor
CHB: Chronic HBV
CMV-IE: CMV immediate early gene
CR: Complete remission
cccDNA: Covalently closed circular DNA
CMV: Cytomegalovirus
DC: Dendritic cell
DLBCL: Diffuse large B-cell lymphoma
ER: Endoplasmic reticulum
EBV: Epstein-Barr virus
EBNA: Epstein-Barr virus nuclear antigens
GBM: Glioblastoma
gB: Glycoprotein B
HBcAg: HBV core antigen
HBVsvp: HBV subviral particles
HBxAg: HBV x antige
HNC: Head and neck cancer
HBsAg: Hepatitis B surface antigen
HBV: Hepatitis B virus
HCV: Hepatitis C virus
HCC: Hepatocellular carcinoma
HA: Histocompatibility antigens
HHV-4: Human herpesvirus 4
HHV-5: Human herpesvirus 5
HIV-1: Human immunodeficiency virus type 1
HPV: Human papilloma virus
hTERT: Human telomerase reverse transcriptase
IARC: International Agency for Research on Cancer
KSHV: Kaposi’s sarcoma-associated herpesvirus
LMPs: Latent membrane proteins
LCLs: Lymphoblastic cell lines
MHC: Major histocompatibility complex
MCPV: Merkel cell polyomavirus
NPC: Nasopharyngeal carcinoma
NK: Natural killer
ORR: Objective response rate
ORFs: Open reading frames
OPC: Oropharyngeal cancer
OS: Overall survival
PDX: Patient-derived xenograft
PBSCT: Peripheral blood SCT
JCPV: Polyomavirus JC
pgRNA: Pregenomic RNA
PFS: Progression-free survival
rcDNA: Relaxed circular DNA
+ssRNA: Single positive-sense RNA
scFvs: Single-chain antibodies
TCR: T cell receptor
TRAC: TCR alpha chain
TAA: Tumor associated antigen
TIL: Tumor-infiltrating lymphocyte
VST: Virus-specific T cell
